# Man, Machine, or Both? What Should Count as an Advance in Dermoscopy

**DOI:** 10.3390/cancers18172742

**Published:** 2026-08-24

**Authors:** Dana N. Pham, Warren H. Chan, Jade Conway, Iman Ali, Clay J. Cockerell

**Affiliations:** 1 Orlando College of Osteopathic Medicine, Winter Garden, FL 34787, USA; dpham039@students.ocom.org; 2 Reveal Research Institute, Dallas, TX 75235, USA; 3 Texas Health Dallas, Dallas, TX 75231, USA; 4 The Departments of Dermatology and Pathology, The University of Texas Southwestern Medical Center, Dallas, TX 75390, USA; 5 Lake Granbury Medical Center, Dallas, TX 75235, USA

## Abstract

New technologies in dermoscopy are often judged by whether they outperform clinicians on selected images. This Editorial argues that a meaningful advance should instead improve care in real clinical settings. Evidence from artificial intelligence, reflectance confocal microscopy, site-specific dermoscopy, and teledermoscopy suggests that the greatest gains occur when technology is integrated with trained clinical judgment, validated prospectively, tested across skin types and anatomical sites, and evaluated for access and patient-relevant outcomes. The central question is not whether a person or machine performs better alone, but whether the combination helps identify skin cancer earlier while avoiding unnecessary biopsies.

## 1. The Case for the Machines, Stated Fairly

Dermoscopy has spent three decades earning its place through evidence. Pooled across clinical studies, it carries a relative diagnostic odds ratio for melanoma of 15.6 over naked-eye examination [1], and a Cochrane review confirmed the benefit in both in-person and image-based evaluations [2]. This track record is exactly why the current moment deserves a careful eye. As artificial intelligence, reflectance confocal microscopy, optical coherence tomography, and teledermoscopy arrive together, it is worth asking a plain question: What should actually count as an advance?

The reflexive answer is a leaderboard. A new algorithm or device is announced, its accuracy is compared against dermatologists, and a headline follows. We want to argue that the leaderboard is the wrong scoreboard, and that the real advances in this field share a different signature.

The strongest version of the “AI is winning” argument is genuinely strong, and we should not wave it away. Esteva and colleagues showed that a single convolutional neural network could classify skin lesions at the level of board-certified dermatologists [3]. Haenssle and colleagues went further, pitting a network against 58 dermatologists and finding that, at matched sensitivity, the network achieved a higher specificity—82.5% versus 71.3%—which, in principle, means fewer unnecessary excisions [4]. Reader studies have since repeatedly found algorithms performing at or above the level of experienced clinicians on curated dermoscopic images [5]. If the goal is to sort a stack of standardized photographs, the machines are, on average, very good.

## 2. Why This Is Not Yet the Same as a Clinical Advance

Here is where we would urge caution. A reader study measures performance on lesions that have already been selected, cropped, and confirmed. The clinic does not work that way. The lesion in front of you is unselected, the lighting is imperfect, and the diagnosis you are trying not to miss may look nothing like the training data.

Three facts should keep us honest. First, public dermatology image datasets substantially overrepresent lighter skin, and diagnostic accuracy has been shown to decline for higher Fitzpatrick phototypes, prompting the development of datasets designed specifically to measure and narrow that gap [6]. Second, regulators now say so out loud: in its 2024 authorization of the first AI-based skin cancer detection device for primary care, the FDA explicitly flagged limited sensitivity data for Fitzpatrick IV to VI skin and advised clinical caution [7]. Third, and most telling, the best real-world results have not come from the algorithm replacing the clinician. Tschandl and colleagues showed that human–computer collaboration outperformed either alone, and that the largest gains accrued to less experienced readers [8]. The machine did not win. The partnership did.

## 3. The Same Lesson, Across the Whole Issue

Once you look for it, the pattern recurs across the topics covered by this Special Issue, not just in artificial intelligence.

Consider reflectance confocal microscopy. Its value in the randomized data is real: adding RCM to the workup of suspect lesions raised positive predictive value from 18.9% to 33.3%, lowered the benign-to-malignant excision ratio from 3.7:1 to 1.8:1, and cut the number needed to excise by 43.4% [9]. But RCM delivers that value as an adjunct to dermoscopy in equivocal lesions, not as a replacement for it [10]. The advance is integration, not substitution.

Consider site-specific dermoscopy. On acral skin, the parallel ridge pattern remains the most useful single criterion for melanoma, and algorithms built on it are still being validated today [11]. No general-purpose classifier absolves a clinician of knowing that the rules change on the palms, soles, face, and nail unit. The advance is expertise applied where the usual patterns fail.

Consider teledermoscopy. Its promise is not diagnostic wizardry; it is access, moving expert-level interpretation to primary care and to places without a dermatologist. The advance here is reach.

In every case, the technology that matters is the one that becomes folded into a workflow and a trained judgment, not the one that posts the highest number in isolation.

## 4. What We Would Ask of the Work in This Issue

For readers deciding what to submit, and for clinicians deciding what to believe, we would offer a few tests.

1. **Prefer prospective, in-workflow validation over reader studies.** A tool that helps a real clinician decide about a real, unselected lesion is worth more than one that tops a benchmark on curated images.
2. **Demand equity data, not equity language.** Ask for performance broken out by Fitzpatrick phototype and anatomic site. A tool that has not been tested on the skin in front of you has not been tested.
3. **Report the failures.** The most useful papers will state where the method breaks, whom it underserves, and what it should not yet be used for, with the same rigor they bring to their successes.
4. **Measure the partnership, not just the machine.** If an algorithm helps a beginner reach an expert’s accuracy, that is a larger contribution than a marginal gain in standalone performance.

## 5. The Advance Worth Making

We are optimistic about where dermoscopy is going, and this optimism is the reason for the caution. The instrument has always rewarded discipline over enthusiasm. The next decade will add powerful new optics and powerful new algorithms, but the advances that endure will be the ones that make a trained clinician better, on every patient, in the ordinary clinic. Man or machine is the wrong question. The right one is whether the two together can identify skin cancer earlier and spare patients the biopsies they never needed. On that question, the evidence is still being written, and this Special Issue is an invitation to write it well.

## Data Availability

No new data were created or analyzed in this Editorial.
